# Spatial Distribution of Incident Pediatric Burkitt Lymphoma in Central and Northern Malawi and Association With Malaria Prevalence

**DOI:** 10.1200/GO.22.28000

**Published:** 2022-05-05

**Authors:** Yolanda Gondwe, Nmazuo Ozuah, Satish Gopal, Tamiwe Tomoka, Yuri Fedoriw, Kate Westmoreland

**Affiliations:** ^1^ University of North Carolina Project Malawi, Lilongwe, Malawi; ^2^ Baylor College of Medicine, Houston, TX; ^3^ National Cancer Institute, Rockville, MD

## Abstract

Burkitt lymphoma (BL) accounts for 90% of pediatric lymphomas in sub-Saharan Africa. Plasmodium falciparum (Pf) malaria is considered an etiological factor of BL. We describe the geographic distribution of pediatric BL in Malawi and association with malaria prevalence (PfPR). We also examine the influence of travel time to cancer referral center as a potential confounder.

**METHODS:**

We enrolled pathologically confirmed incident pediatric BL cases (2013-2018) into a prospective observational cohort at Kamuzu Central Hospital (KCH) in Lilongwe, the main tertiary cancer referral center serving central and northern Malawi. District of residence was recorded for all participants. We calculated district-level average annual BL incidence rate (BLIR) using census population estimates. PfPR was extracted from the National Malaria Control Program 2010 report. BLIR and PfPR maps were constructed in QGIS. Moran's I was used to identify BL spatial clusters. Travel time to KCH was modeled in AccessMod. Pearson's correlation and multivariate regression was used to statistically examine the relationship between PfPR, travel time to KCH and BL.

**RESULTS:**

Of 220 participants, 69 (31%) resided in Lilongwe. BLIR was higher in central region districts (0.86 cases per 100,000) than northern districts (0.32 cases per 100,000). BLIR was elevated in lakeshore districts. Districts with elevated PfPR tended to have elevated BLIR. A low-risk BL cluster was detected in the northern region. BLIR was lower in districts where travel time to KCH exceeded 4 hours, particularly the north. Statistically, BLIR was positively correlated with PfPR (*r* = 0.77, *P* < .01) and negatively correlated with travel time to KCH (*r* = –0.67, *P* < .01). A 1% increase in PfPR predicted an increase in BLIR of 0.02 cases per 100,000 (*P* = .03) when controlling for travel time to KCH.

**CONCLUSION:**

Our study supports evidence for an association between Pf and BL. Additionally, our results may indicate a need to improve geographic accessibility to tertiary cancer services in Malawi's northern region.

